# Radiotherapy for T3N0 glottic carcinoma without cord fixation: elective nodal irradiation or not?

**DOI:** 10.18632/oncotarget.19337

**Published:** 2017-07-18

**Authors:** Ryo Toya, Ryuji Murakami, Daizo Murakami, Tetsuo Saito, Tomohiko Matsuyama, Yutaka Toya, Yasuyuki Yamashita, Natsuo Oya

**Affiliations:** ^1^ Department of Radiation Oncology, Kumamoto University Hospital, Kumamoto, Japan; ^2^ Department of Medical Imaging, Faculty of Life Sciences, Kumamoto University, Kumamoto, Japan; ^3^ Department of Otolaryngology-Head and Neck Surgery, Kumamoto University Hospital, Kumamoto, Japan; ^4^ Department of Diagnostic Radiology, Kumamoto University Hospital, Kumamoto, Japan

**Keywords:** glottic carcinoma, radiotherapy, lymph node metastasis, elective nodal irradiation, clinical target volume

## Abstract

**Background:**

Although the T3 category has been changed in the sixth edition of the TNM staging system proposed by the Union for International Cancer Control (UICC), the appropriate clinical target volume (CTV) of elective nodal irradiation (ENI) for T3N0 glottic carcinoma without cord fixation, which was formerly treated as a T1-2N0 disease, is not fully discussed.

**Materials and Methods:**

We retrospectively analyzed 64 patients staged or restaged as T3N0 disease without cord fixation. All patients received irradiation to the primary lesion alone using opposed lateral fields. Surgery was performed in 10 patients without tumor regression after the delivery of 40 Gy. The other 54 patients received a median total dose of 66 Gy. Concurrent chemoradiotherapy (CRT) with low-dose cisplatin and UFT (low-dose CRT) and docetaxel, cisplatin, and 5-fluorouracil (TPF-CRT) were performed in 23 and 19 patients, respectively.

**Results:**

Eighteen (28.1%) patients suffered treatment failure; all were recorded as local failure alone. The 5-year local control rates for RT alone, low-dose CRT, and TPF-CRT groups were 51.7%, 61.6%, and 93.8%, respectively (*p* = 0.027). The 5-year laryngeal preservation rates for RT alone, low-dose CRT, and TPF-CRT groups were 57.4%, 81.6%, and 89.5%, respectively (*p* = 0.048).

**Conclusions:**

The rate of regional failure was zero when irradiating the primary lesion alone using opposed lateral fields. This treatment technique covers the most level III regions; hence, CTV for ENI should include level III alone.

## INTRODUCTION

Glottic carcinoma is the most common laryngeal cancer [1]. Radiotherapy (RT) with or without chemotherapy is one of the recommended therapeutic approaches for the treatment of T3 disease [1, 2]. After the sixth edition of the TNM staging system was proposed in 2002 by the Union for International Cancer Control (UICC), T3 glottic carcinoma included paraglottic space invasion and/or minor thyroid cartilage erosion in addition to vocal cord fixation, which was listed in the fifth edition. Subsequently, no essential revision was made, and in the newest eighth edition of the TNM staging system, T3 glottic carcinoma is defined as “tumor limited to larynx with vocal cord fixation and/or invades paraglottic space and/or inner cortex of the thyroid cartilage.”

As lymph node (LN) metastasis is extremely rare, T1-2N0 glottic carcinoma has been treated with RT without elective nodal irradiation (ENI) [3]. However, ENI for levels II–IV has been traditionally recommended for T3N0 disease [4]. Although the T3 category has changed, the optimal clinical target volume (CTV) of ENI for T3N0 disease without cord fixation, which was formerly treated as a T1-2N0 disease, is not fully discussed. Before the introduction of the UICC sixth edition, we reported the existence of the invasion of the paraglottic space and inner cortex of the thyroid cartilage [5, 6]. In the present study, we investigated the optimal CTV of ENI for T3N0 disease without cord fixation by evaluating the regional failure based on our hospital’s database.

## RESULTS

**Figure 1:**
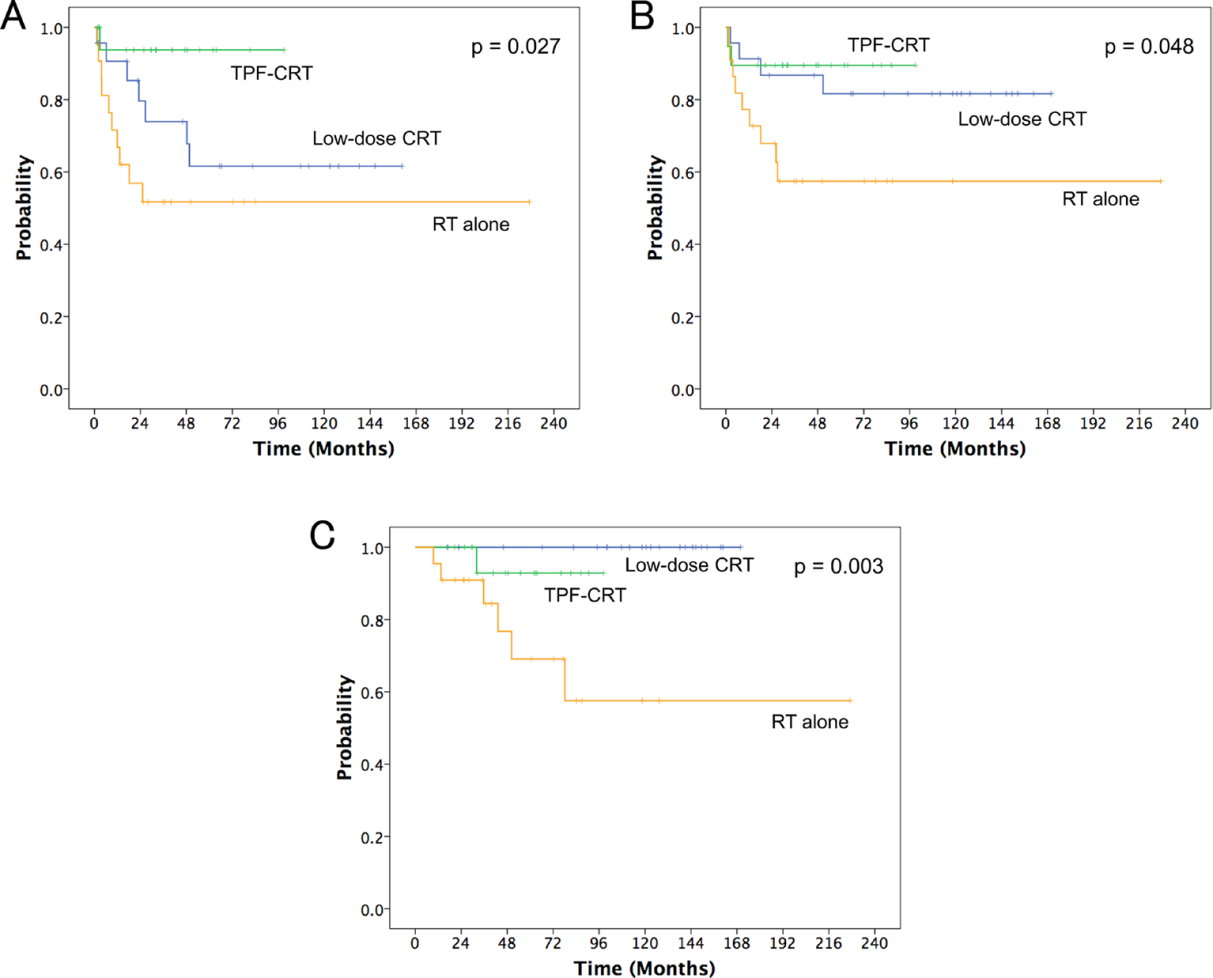
(**A**) local control, (**B**) laryngeal preservation, and (**C**) overall survival curves for the three treatment groups of radiotherapy (RT) alone, low-dose chemo-RT (CRT) with low-dose cisplatin and UFT (low-dose CRT), and concurrent CRT with docetaxel, cisplatin, and 5-fluorouracil (TPF-CRT).

Figure 1 shows the local control (LC), laryngeal preservation (LP), and overall survival (OS) curves for patients with T3N0 glottic carcinoma without cord fixation treated using RT with or without chemotherapy over 240 months. The median follow-up duration was 69.3 months (range: 9.5–227.1 months). During the follow-up period, 46 (71.9%) patients experienced tumor control and 18 (28.1%) suffered treatment failure. All patients with treatment failure were recorded as local failure alone; regional and distant failure was not observed. Of the 18 patients with local failure, LP was obtained with salvage surgery in 10 patients. Overall, LP was obtained in 49 (76.6%) patients. Two patients with local failure underwent neck dissection, which was confirmed as pathologically node-negative.

The 5-year LC rates for all patients were 64.9%. The 5-year LC rates for RT alone; concurrent chemo-RT (CRT) with low-dose cisplatin and UFT (low-dose CRT group); and concurrent CRT with docetaxel, cisplatin, and 5-fluorouracil (TPF-CRT group) were 51.7%, 61.6%, and 93.8%, respectively (*p* = 0.027, Figure 1). The 5-year LP rates for all patients were 74.9%. The 5-year LP rates for RT alone, low-dose CRT, and TPF-CRT groups were 57.4%, 81.6%, and 89.5%, respectively (*p* = 0.048, Figure 1). The 5-year OS rates for all patients were 88.4%. The 5-year OS rates for RT alone, low-dose CRT, and TPF-CRT groups were 69.1%, 100%, and 92.9%, respectively (*p* = 0.003, Figure 1).

All toxicities were manageable, and there were no grade 5 events. One patient experienced grade 4, six patients experienced grade 3, and eight patients experienced grade 2 myelosuppression. One patient experienced grade 3 liver toxicity. Thirteen patients experienced grade 2 gastrointestinal toxicities. Four and two patients experienced grade 2 mucositis and dermatitis, respectively.

## DISCUSSION

The results of our study revealed that the rate of regional failure when irradiating primary lesions alone using opposed lateral fields for patients with T3N0 glottic carcinoma without cord fixation was zero. The traditionally recommended CTV for ENI was based mainly on pathological examinations using palpation rather than based on imaging. In pathological analyses of the early 1990s, the incidence of LN metastasis for clinical N0 patients was reported as more than 30% [7]. However, the introduction of modern imaging techniques such as computed tomography (CT), magnetic resonance imaging (MRI), and 2-deoxy-2-[18F]fluoro-D-glucose (FDG)-positron emission tomography (PET) into the nodal staging of head and neck cancer has provided excellent diagnostic accuracy [8, 9]. Furthermore, pathological studies revealed that level III is the most commonly involved cN0 glottic carcinoma regions [7, 10]. As irradiation with opposed lateral fields, which is typically used for early T1–T2N0 glottic carcinoma, covers most of the level III regions, microscopic metastasis to this level should be electively irradiated [3, 11]. Therefore, CTV for ENI should include level III alone for T3N0 glottic carcinoma without cord fixation. Hence, irradiation to the primary lesion alone using opposed lateral fields was an appropriate treatment technique for this disease. Prior to the revision of UICC sixth edition, Pelliteri et al. [12] treated 162 T1-2N0 glottic carcinoma patients with total RT dose of 60–70 Gy using this technique. They investigated the pattern of failure and found that there was no patient with regional failure alone. Yamazaki et al. [13] performed a prospective randomized study for T1N0 glottic carcinoma using the same technique. One hundred and eighty patients were randomly allocated to either the treatment arm of total RT dose of 60–66 Gy in 2-Gy fractions or total RT dose of 56.25–63 Gy in 2.25-Gy fractions. They reported that no regional failure was observed before local failure in both treatment arms. Jones et al. [14] treated 115 patients with T1-2N0 glottic carcinoma patients with a total RT dose of 60.75–75.6 Gy using a similar technique; they irradiated the primary lesion alone with the 3-field technique delivering 90% of the total dose using opposed lateral fields with the remainder of the dose coming from an anterior field. They found no isolated regional recurrence and recommended against ENI for these diseases.

In previous reports of treatment results with RT alone for the T3 disease, the LC, LP, and OS rates were 35%–65%, 60%–75%, and 45%–50%, respectively [15–18]. In the results of the RTOG 91-11 trial, the LC, LP, and OS rates for stage III–IV laryngeal cancer were 53.6%, 65.8%, and 53.8% for RT alone group and 71.1%, 83.6%, and 55.1% for concurrent CRT group, respectively [19]. Al-mamgani et al. [20] treated 170 T3 laryngeal cancer patients using RT with or without concurrent chemotherapy. The LC and OS rates were 68% and 49%, respectively. Our treatment results were comparable to those of these previous reports with acceptable toxicities.

Our study has some limitations, including the retrospective design, relatively small number of patients for a long study period, and variability in RT doses and chemotherapy regimens. We could not rule out the potential influence of the surgical intervention after the delivery of 40 Gy on treatment outcomes of regional failure. The chemotherapy regimens were relatively uncommon in the current practice. We analyzed the pattern of failure based on patients treated with RT using opposed lateral fields. Recent planning studies suggested the dosimetric benefit of intensity-modulated RT (IMRT) for carotid artery sparing in the treatment of early glottic carcinoma [21]. Although we recommend ENI for level III alone, we cannot comment on the laterality of this level.

## MATERIALS AND METHODS

### Patients

This retrospective study was approved by the institutional review board of our hospital. Informed consent was obtained from all patients prior to treatment. Between November 1992 and February 2015, 515 patients with pathologically diagnosed glottic carcinoma who had undergone pretreatment physical, endoscopic, and radiological examinations were treated with RT with or without chemotherapy. Of these, 74 patients were staged or restaged as T3N0 without cord fixation according to the eighth edition of the UICC; 47 patients had T1-2N0 with invasion of the paraglottic space and/or inner cortex of the thyroid cartilage according to the UICC fifth edition and 27 patients had T3N0 without cord fixation according to the UICC sixth or seventh edition. Finally, 64 patients were included in this study and 10 patients were excluded from analysis; eight patients received ENI before 1999, one patient had coexisting advanced small cell lung cancer, which required simultaneous CRT, and one patient was lost to follow-up within 6 months. All patients were males and ranged in age from 43 to 88 years (median age: 69 years) at the start of their treatment.

### Radiological examinations

Pretreatment examinations included ultrasonography (US), MRI, CT, and/or FDG- PET/CT. MR studies were also performed with 40 Gy irradiation for the interim assessment of the tumor regression [22].

### Treatment

Between 1992 and 1998, all 9 patients were treated with RT alone. Between 1999 and 2005, we performed concurrent low-dose CRT with low-dose cisplatin and UFT in 23 patients [6]. To receive this treatment, the patients were required to be 20–80 years of age, have an Eastern Cooperative Oncology Group performance status (PS) of 0–2, a life expectancy of at least 3 months, adequate hematolotic (WBC ≥ 4000/mm^3^, Hb ≥ 10 g/dl, Plts ≥ 100 000/mm^3^) and renal (Cr ≤ 1.2 mg/dl, CCr ≥ 60 ml/min) function, no severe complications, and provide informed consent. The patients received an intravenous infusion of cisplatin (4 mg/m^2^) for 1 h prior to delivery of the daily fraction and orally took the fixed dose of UFT (150 mg of tegafur three times a day; 450 mg daily) after meals starting on the first day of irradiation and continuing for 4 weeks. Nine patients received RT alone in this period because they did not meet the eligibility criteria.

Between 2006 and 2015, we performed concurrent TPF-CRT with docetaxel, cisplatin, and 5-fluorouracil in 19 patients [23]. For inclusion, patients had to be 20–80 years of age, have a PS of 0–2, a life expectancy of at least 3 months, adequate hematolotic (WBC ≥ 3500/mm^3^, ANC ≥ 2000/mm^3^, Hb ≥ 9.5 g/dl, Plts ≥ 100 000/mm^3^), renal (Cr ≤ 1.2 mg/dl, CCr ≥ 60 ml/min), and hepatic (TB 1.5 mg/dl, AST ≤ 2 × ULN, ALT ≤ 2 × ULN) function, no severe complications, and provide informed consent. The patients received an intravenous infusion of docetaxil (50 mg/m^2^) for 1 h on day 1. More than 1 h after completing the docetaxel infusion, 5-FU (600 mg/m^2^/day) was delivered by continuous intravenous infusion on days 1–5. Cisplatin (60 mg/m^2^) was given intravenously on day 4. Two cycles of chemotherapy were repeated every 4 weeks. Four patients received RT alone in this period because they did not meet the eligibility criteria.

**Figure 2:**
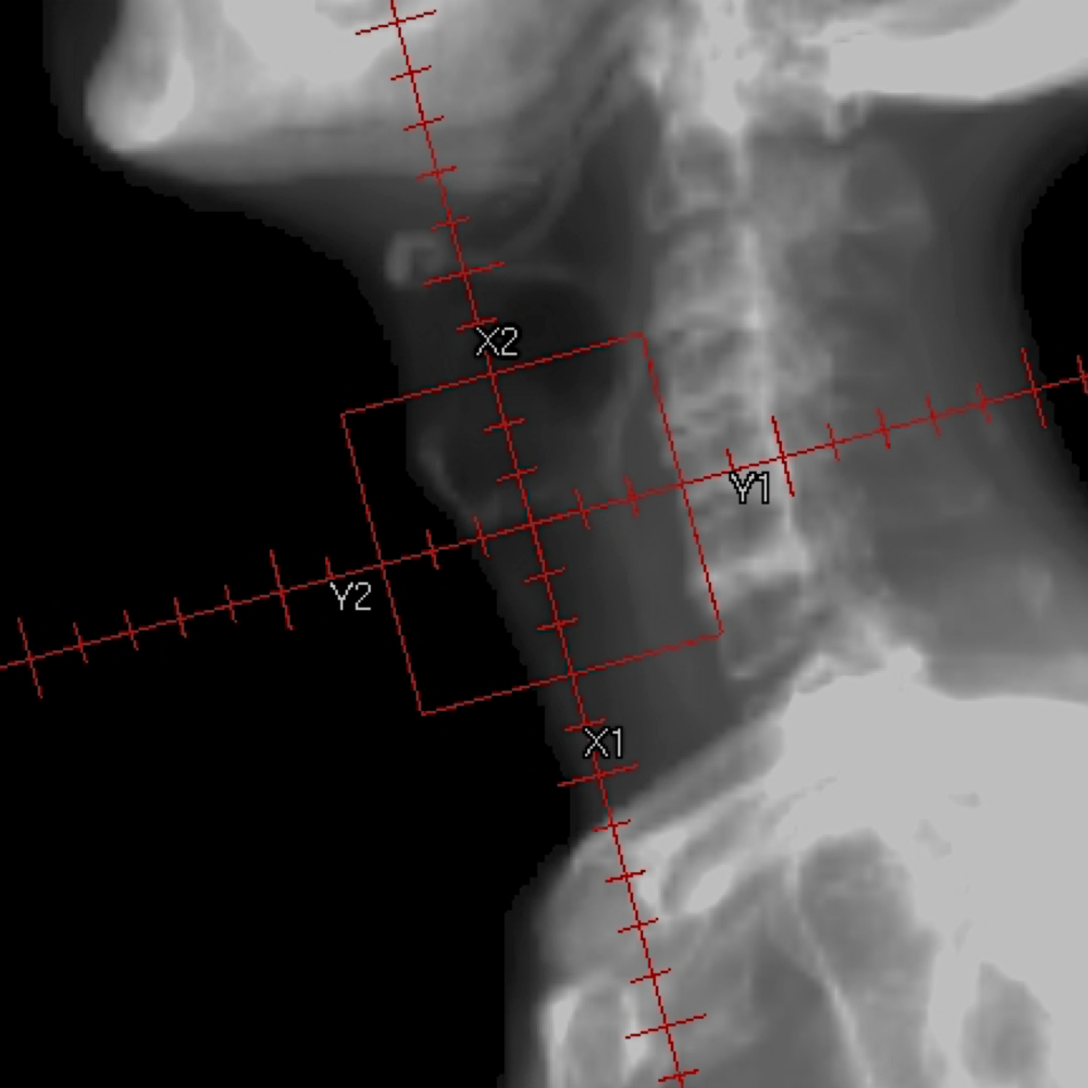
Radiotherapy field for the T3N0 glottic carcinoma without cord fixation

RT was delivered using a 3–4 MV linear accelerator in daily fractions ranging from 1.8 to 2 Gy. All 64 patients received irradiation to the primary lesion alone with opposed lateral fields (Figure 2). RT field extended from the thyroid notch superiorly to the inferior border of the cricoid to 1cm behind the posterior aspect of the thyroid cartilage with a 1-cm fall-off anteriorly [11, 24]. Field borders were modified for each patient depending on the anatomical extent of the particular lesion. The median field size was 36 cm^2^ (range: 25–49 cm^2^, corresponding to 5 × 5–7 × 7 cm). After the delivery of 40 Gy, otolaryngologists and radiation oncologists, by consensus, performed interim assessment to evaluate tumor regression from endoscopic and radiological examinations [6, 22]. In 10 patients, the tumors showed no regression and surgery was performed: total laryngectomy (*n* = 4) and laryngeal preservation surgery (*n* = 6). The remaining 52 patients received a median total dose of 66 Gy (range: 60–72 Gy); a median total dose of 70 Gy (range: 63.8–72 Gy) for RT alone, 68 Gy (range: 60–72 Gy) for low-dose CRT, and 66 Gy (range: 64–66 Gy) for TPF-CRT groups, respectively.

### Follow-up

After the completion of treatment, patients were evaluated every 1–2 months during the first year, every 3 months during the second year, and every 6 months thereafter. All patients underwent physical and endoscopic examinations at each follow-up visit. When a follow-up endoscopy detected a suspicious lesion, biopsy specimens were examined to rule out tumor recurrence. Post-treatment US, MR imaging, and/or CT studies were also performed within 1–2 months after the completion of treatment and every 6 months thereafter or when clinically indicated. A diagnosis of treatment failure was based on the pathology specimen results. Failures were classified as involving the primary lesion (local), neck LN metastases (regional), or distant metastases (distant). Treatment-associated toxicities were graded according to the Common Terminology Criteria of Adverse Events Version 4.0 (CTCAE V4.0).

### Statistical analysis

LC, LP, and OS rates were calculated from the initiation of treatment using the Kaplan–Meier method. Patients who underwent surgery and had a pathological complete response to the treatment were censored at the time of surgery for the calculation of LC according to Mendenhall et al. [16]. The effect of chemotherapy was evaluated using log-rank statistics. Differences with *p*-values < 0.05 were considered statistically significant. Statistical calculations were performed using SPSS software, version 24.0 (SPSS Inc., Chicago, IL, USA).
